# Inferring China’s excess mortality during the COVID-19 pandemic using online mourning and funeral search volume

**DOI:** 10.1038/s41598-023-42979-1

**Published:** 2023-09-20

**Authors:** Li Huang, Oliver Zhen Li, Ximing Yin

**Affiliations:** 1 Shanghai Jiao Tong University, Shanghai, China; 2 Shanghai Lixin University of Accounting and Finance, Shanghai, China; 3 National University of Singapore, Singapore, Singapore; 4 Hunan University, Changsha, China

**Keywords:** Human behaviour, Epidemiology, Health policy, Public health

## Abstract

We construct a mourning and funeral index, using online search volume for “wreath and elegiac couplet”, “obituary”, “mortuary house”, “cinerary casket”, “cremation” and “pass away”, to infer excess cases of mortality in China during the COVID-19 pandemic. During the 3-month period (December 2022–February 2023) after China ended its Zero-COVID policy, there were around 712 thousand excess cases of mortality. These excess cases of mortality, bench marked against the 2-year period preceding the pandemic, could be directly or indirectly related to COVID-19. During the 35-month Zero-COVID regime (January 2020–November 2022), the excess death toll was a negative 1480 thousand. Overall, by delaying the surge in infections, China might have saved 767 thousand lives. While these estimates are based on various assumptions and can be imprecise, China’s COVID-19 experience could reasonably be characterized by a sharp surge in deaths after its departure from Zero-COVID and a steady pattern of lives saved during the Zero-COVID regime.

## Introduction

China implemented stringent control policies aimed at protecting public health along the trajectory of the COVID-19 pandemic, including a national emergency response to contain its initial outbreak in Wuhan in early 2020 and the subsequent adoption of a nationwide “Zero-COVID” policy^1,2^. This policy had been effective in keeping China’s COVID-19 death toll among the lowest in the world before December 2022^3^. However, prolonged control measures, in addition to their costly implementation, became infeasible when economic recovery and civil liberty were severely compromised^4–6^. On the other hand, a sudden departure from Zero-COVID could carry a huge human life toll^7,8^.

On December 7, 2022, China moved away from zero-COVID. Cases of infections and deaths soared^9,10^. Officially recorded deaths in hospitals peaked on the 5th January and receded towards zero in mid-February^11^. In-hospital mortality figure released by the Chinese government stood at 83,150 from December 8th, 2022 to February 9th, 2023. However, controversies are centered on whether China’s reported death toll in hospitals based on a narrow definition of COVID-19 deaths can under-represent a more realistic picture^12^.

We devise a simple yet intuitive approach to infer China’s death toll during January 2020–February 2023 using Internet search queries on mourning and funeral terms from China’s dominant search engine, Baidu. Data on web search queries are available on a daily basis and can provide timely and broad-reaching disease surveillance complementary to traditional methods^13^. While information on country and province-level cases of mortality is only disclosed officially by the Chinese authority once a year and that the most recent disclosure was for 2020, we can estimate daily/month excess cases of mortality based on reported mortality prior to the pandemic plus a statistical association between web search volume of mourning and funeral terms and cases of mortality.

Baidu web search volume is a reasonable measure for the general population’s need for collecting information for mourning and funeral purposes. Web search is a revealed need measure, as people commonly use a search engine to collect useful information. For example, web search data can predict home sales, automotive sales, tourism, unemployment, and crises in the financial market^14–16^. People search for global warming related terms in response to experiencing warmer than usual temperatures in their areas^17^. In disease surveillance, search data for terms related to influenza can assist the detection, monitoring, and prediction of flu outbreaks that benefit timely health policymaking^13,18–20^. Web search information has also been used to track and predict the spread of COVID-19^21,22^. In our scenario, incidences of mortality cause people to conduct web searches to gather mourning and funeral related information. Further, Baidu has been the dominant search engine in mainland China. As of March 2022, Baidu accounted for 84.3% of search engine market share in China^23^. With an Internet user penetration ratio in China surpassing 70%^24^, Baidu search volume is likely representative of queries made by the general population of mainland China.

The validity of our estimation is based on the key assumption (which we verify) that web searches for mourning and funeral terms are positively correlated with cases of mortality. Specifically, we obtain daily search volume for “wreath and elegiac couplet”, “obituary”, “mortuary house”, “cinerary casket”, “cremation” and “pass away”. This mourning and funeral search list, though likely not exhaustive, reasonably represents China’s typical mourning and funeral tradition and practices. For example, relatives and friends need to search for information on how to purchase and prepare wreaths and elegiac couplets, on how to write obituaries, information on mortuary houses and the acquisition of cinerary caskets (cremation is almost the only option in China), information on civil and administrative matters related to the deceased. We average search volumes for these six queries and construct a mourning and funeral index. We observe a sharp rise in this index after China’s departure from Zero-COVID in December 2022. Based on this index, we extrapolate daily excess cases of mortality throughout the pandemic trajectory for each of mainland China’s province as well as the country as a whole using pre-pandemic reported cases of mortality as a benchmark.

We first estimate excess cases of mortality during the three-month period (December 2022 to February 2023) after China moved away from Zero-COVID. Benchmarked against pre-COVID-19 years 2018 and 2019 with monthly-mean adjustment of the mourning and funeral index, there were roughly 712 thousand excess cases of mortality during the three-month period. It is important to note that these excess deaths can be directly related to COVID-19 or indirectly related to COVID-19 as a surge in COVID-19 cases can cause a run on hospital resources, leading to deaths when patients with other diseases could not get adequate medical care.

Considering the in-hospital mortality figure released by the Chinese government of 83,150 from December 8th, 2022 to February 9th, 2023, the figure based on our monthly-mean adjusted mourning and funeral index during the same period is 637 thousand (about 7.7 times that of the government figure), which is considerably high. This difference can be due to deaths of all causes outside hospitals and deaths in hospitals that were not directly attributed to COVID-19 according to Chinese authority’s definition. With around one-fourth of all deaths in China occurring in hospitals^25^, our estimation suggests that the increase in the cases of mortality after the end of Zero-COVID was potentially significantly due to deaths outside hospitals.

We then estimate China’s excess cases of mortality during the 35-month (January 2020 to November 2022) Zero-COVID regime. With monthly-mean adjustment of the mourning and funeral index, there were around 1,480 thousand lives saved during this period. Overall, by delaying the surge in infections, China might have saved 767 thousand lives.

We admit that our estimation relies on assumptions that may or may not hold and that the figures we infer from the mourning and funeral index can be imprecise. However, it can be reasonably concluded that China’s COVID-19 experience is characterized by a sharp surge in deaths after it moved away from Zero-COVID as well as a steady pattern of lives saved during the Zero-COVID regime.

## Results

### Time-series monthly-mean adjusted estimation

#### During post-zero-COVID regime (December 2022–February 2023)

**Figure 1:**
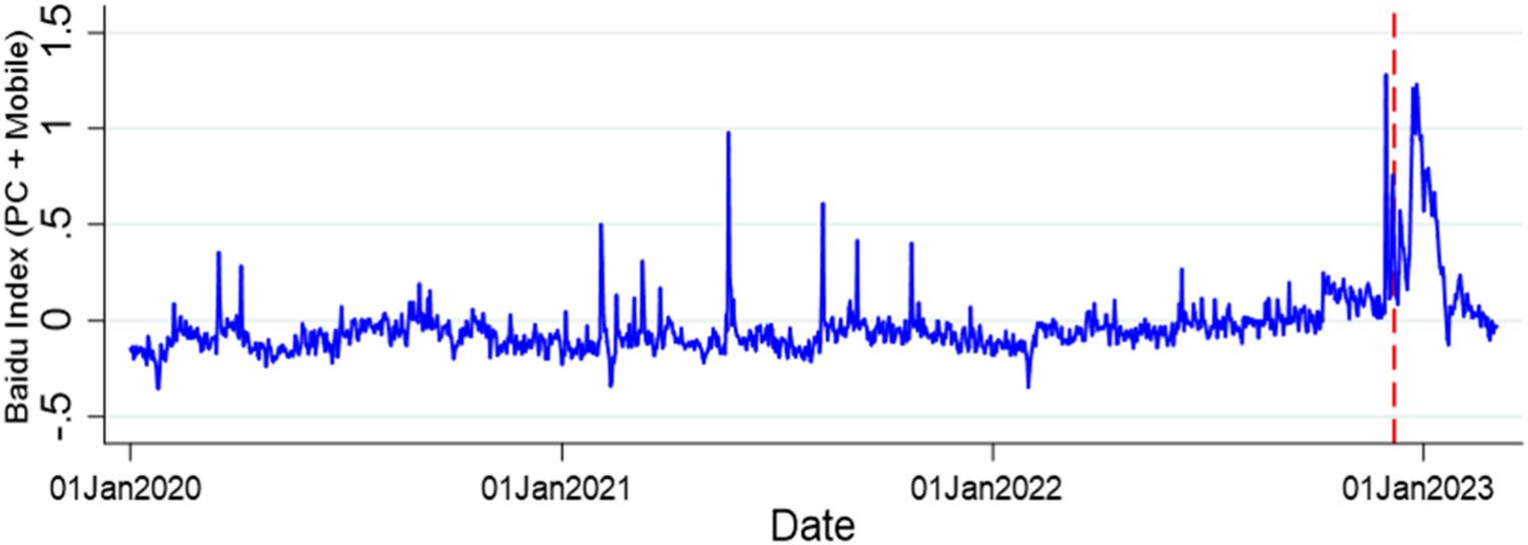
Mourning and funeral index, based on monthly-mean adjustment during January 1, 2020 to February 28, 2023.

Figure 1 shows the national-level monthly-mean adjusted mourning and funeral index which is the population weighted average of each province’s index during January 2020 to February, 2023. There is a significant increase in this index during the three-month period. China’s departure from zero-COVID in early December 2022 was significantly and immediately associated with an increase in web searches for mourning and funeral terms. This surge tapered off in February, 2023. The mourning and funeral index appears to be synchronous to the pandemic’s trajectory in China around the policy shift.

**Figure 2:**
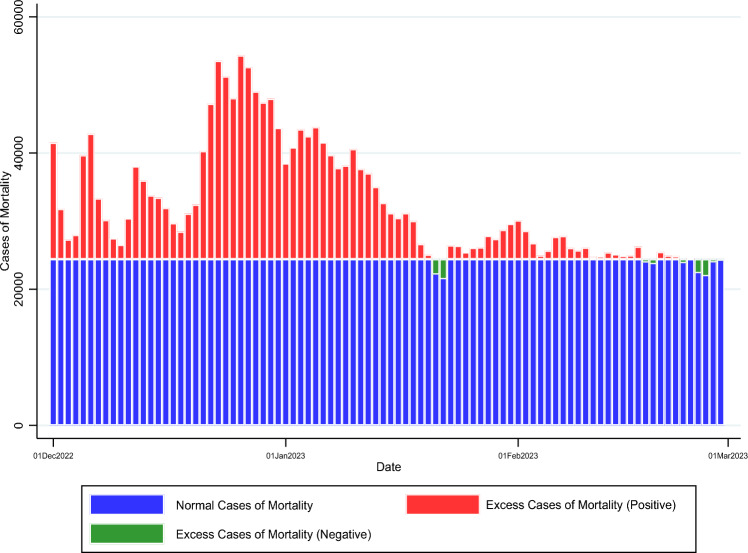
National Daily Excess Cases of Mortality during December 2022 to February 2023, Based on Monthly-Mean Adjusted Mourning and Funeral Index.

**Figure 3:**
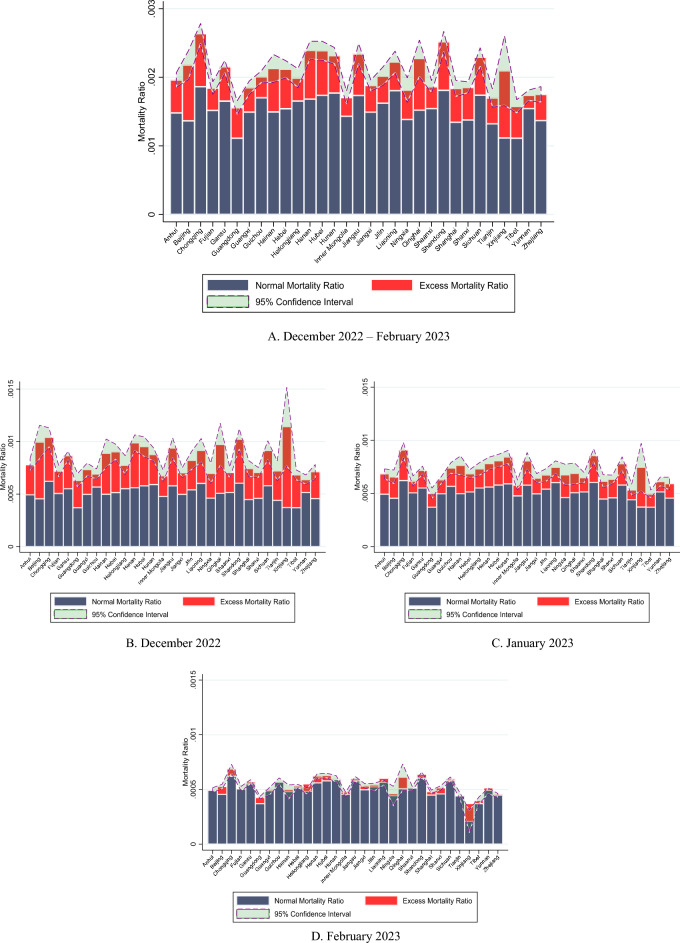
Excess mortality ratio by provinces during December 2022 to February 2023, based on monthly-mean adjusted mourning and funeral index.

We then use the monthly-mean adjusted mourning and funeral index to infer excess cases of mortality. Inferred excess cases of mortality during December 2022 to February 2023 are presented in Table S1. Shandong has the highest number of excess cases of mortality (76,838, 10.28% of the national total), followed by Henan (70,197, 9.85% of the national total) and Guangdong (56,363, 7.91% of the national total). Note that these are three provinces with a large population. Summing up thirty-one provinces, we arrive at the national figure of 712,905 (432,519 for December 2022; 254,573 for January and 25,812 for February 2023). These excess cases of mortality could be attributable to deaths directly or indirectly associated with COVID-19 after the departure from Zero-COVID. Figure 2 presents a visual of national daily excess cases of mortality. If there is no significant lead or lag time between cases of mortality and the search volume for mourning and funeral terms, then it appears that deaths peaked in late December 2022 and early January 2023. Towards the end of January, this wave of deaths considerably subsided. Table S3 presents the numbers for each province and Fig. 3 presents a visual of province-level excess mortality ratio statistics for the three-month period as a whole (Fig. 3A) and the three months separately (Fig. 3B–D).

#### During zero-COVID regime (January 2020–November 2022)

**Figure 4:**
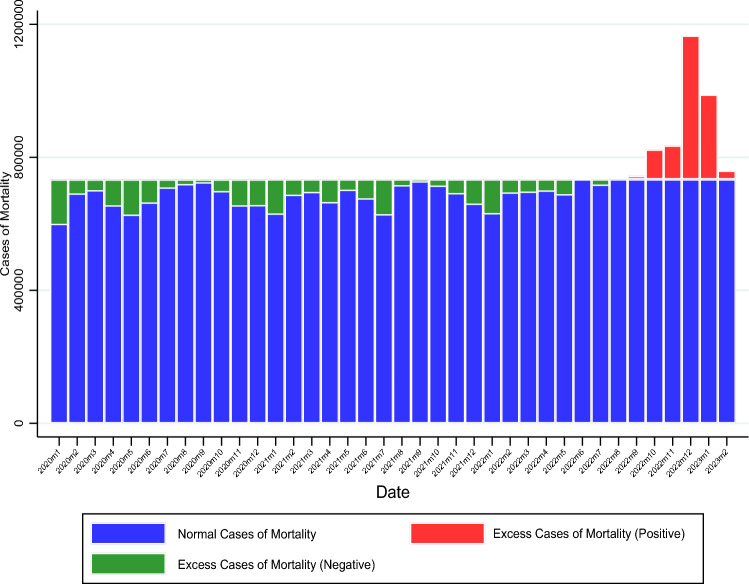
Monthly excess cases of mortality from January 2020 to February 2023, based on monthly-mean adjusted mourning and funeral index.

While there was a steep increase in excess cases of mortality during December 2022 to February 2023 after China moved away from Zero-COVID, for thirty out of the thirty-five months during the Zero-COVID regime (January 2022 to November 2022), excess cases of mortality were negative for mainland China (Fig. 4). Excess cases became positive and significant during October and November of 2022 which saw wide spread infections that potentially led to the decision to move way from Zero-COVID. From Table 1, the total number of excess cases of mortality during January 2020 to November 2022 was a negative 1,480,900. Therefore, strict measures and their induced human behaviour changes could have potentially reduced deaths unrelated to COVID-19^26^.

**Table 1:** National monthly excess cases of mortality from January 2020 to February 2023, based on monthly-mean adjustment of the mourning and funeral index and regression estimation.

Year	Month	Excess cases of mortality
2020	1	− 137,527
2020	2	− 46,040
2020	3	− 36,174
2020	4	− 81,779
2020	5	− 109,612
2020	6	− 73,277
2020	7	− 27,914
2020	8	− 17,902
2020	9	− 12,629
2020	10	− 39,090
2020	11	− 81,708
2020	12	− 81,144
2021	1	− 106,414
2021	2	− 49,861
2021	3	− 41,715
2021	4	− 72,203
2021	5	− 34,967
2021	6	− 60,816
2021	7	− 108,585
2021	8	− 21,263
2021	9	− 9590
2021	10	− 22,515
2021	11	− 45,049
2021	12	− 76,533
2022	1	− 105,462
2022	2	− 43,435
2022	3	− 40,853
2022	4	− 37,366
2022	5	− 48,708
2022	6	1429
2022	7	− 19,619
2022	8	6460
2022	9	10,050
2022	10	89,359
2022	11	101,551
2022	12	432,519
2023	1	254,573
2023	2	25,813

#### Overall tally (January 2020–February 2023)

It appears that China’s COVID-19 experience can be characterized by a surge in deaths after it moved away from Zero-COVID as well as a steady pattern of lives saved during the Zero-COVID regime. Overall, by delaying the surge in infections with the Zero-COVID policy, China might have saved 767,955 lives (Table 1).

### Out-of-sample validity and elasticity of mortality with respect to the mourning and funeral index

#### Out-of-sample validity

An important assumption of our analysis is that web-search for mourning and funeral related terms can mimic actual cases of mortality. From Panel B of Table S2, during 2011–2019, the correlation between cases of mortality and the six search terms ranges from 0.433 (pass away, *p* < 0.01) to 0.644 (wreath and elegiac couplet, *p* < 0.01). The correlation related to the average of the six terms is 0.578 (*p* < 0.01). Our assumption of a positive association between cases of mortality and the mourning and funeral index is thus likely valid.

To determine if we have out-of-sample validity, we obtain raw annual mourning and funeral index for the period 2011–2019, before the pandemic. During 2011–2017, we regress the natural logarithm of annual cases of mortality for 31 provinces on the natural logarithm of annual mourning and funeral index for these provinces plus a quadratic time trend. We use the logarithm form regression in order to obtain the elasticity of cases of mortality with respect to the mourning and funeral index. This will enable us to determine if we can use a percentage increase in the index to infer a percentage change in mortality. We add the time trend to tease out a portion of mortality that can be expected based on a non-linear time trend. We use an annual regression as we only have annual cases of mortality for each province.

**Figure 5:**
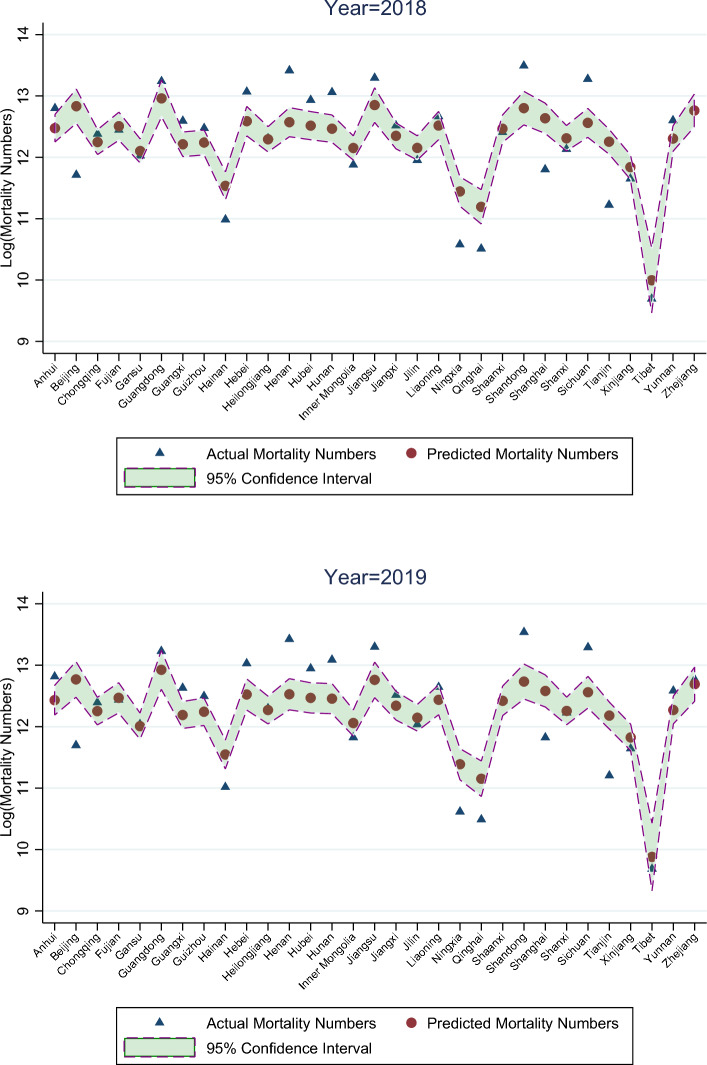
Out-of-sample comparison between estimated and actually cases of mortality.

Tables S4 shows that the coefficient on the index is positive (1.020, *t* = 8.11) and the regression adjusted R-squared is 65.7%. Therefore, the association between mortality and the mourning and funeral index is significant. Based on the regression coefficients, we estimate expected annual logarithm cases of mortality and compare them with actual annual logarithm cases of mortality for all provinces during 2018–2019. The difference is insignificant (*t* = 0.08 for 2018 and *t* = − 0.49 for 2019). Figure 5 provides a visual for this comparison for 2018 and 2019. We can see that the actual numbers scatter around the estimated numbers and are often within the 95% confidence interval of the estimated numbers. It thus appears reasonable that we can use a change in the mourning and funeral index to infer a change in the cases of mortality, with a quadratic time trend already taken care of.

#### Elasticity of mortality with respect to the mourning and funeral index

The natural logarithm regression suggests that the elasticity of cases of mortality with respect to the mourning and funeral index is 1.020, which is not statistically different from one. This implies that a certain percentage change in the mourning and funeral index would translate into an almost identical percentage change in the cases of mortality. This coefficient on the mourning and funeral index that is not statistically different from one justifies our simple and intuitive approach of using monthly-mean adjustment of the mourning and funeral index to infer excess cases of mortality.

## Discussion

Prior to China’s departure from Zero-COVID, researchers were concerned about the potential risk associated with such a departure and forecasted likely casualty scenarios. Medical forecasting models usually impose hypothetical values on key parameters^27,28^. For example, a calibrated model^27^ based on the 2022 Omicron outbreak in Shanghai to project COVID-19 outcome in the absence of non-pharmaceutical interventions suggests about 1.6 million deaths (1.10 deaths per 1000 inhabitants) over a 6-month period. Using experiences of Hong Kong and South Korea as prototypes, researchers suggest a wide range of possible death toll depending on infection rate and extra protections available for the elderly^28^. Uncertainties exist regarding potential changes in dominant variants, health system strain, and the impact on non-COVID-19 deaths. Indeed, prediction models are difficult to develop and subject to wide uncertainties^29^. With historical data, it is nearly impossible to determine precisely whether interventions are sufficient to quell an epidemic or an epidemic will grow unabated^30^. While our inferred cases of mortality is considerably higher than the Chinese authority’s disclosed figure, it is lower than those predicted by medical models.

We do not strive to produce the real death toll. Instead, we offer an estimate of the death toll by combining real time web search traffic and observed empirical patterns. While our estimate is not necessarily precise and the real toll will perhaps never be known, our approach can potentially inform public health policymakers of the likely consequences of certain disease prevention measures and policies.

A few caveats exist for our research. First, our inference relies on the assumption that a steady and consistent portion of people use web searches to gather mourning and funeral related information, over time and across provinces. To the extent that there is a fundamental change in web search habits or a change in the composition of web-search-savvy people, our estimates can have large errors or biases. This caveat is somehow alleviated by a reasonably decent statistical correlation between the index and the actual cases of mortality and out-of-sample validity. Second, our approach also relies on that our list of mourning and funeral terms reflects decently China’s mourning and funeral tradition and practices. To the extent that people in a rapidly modernizing China are changing their traditional ways of mourning and conducting funerals, our estimation can again have biases. Third, the COVID-19 situations and the related government quarantine initiatives can potentially alter people’s mourning and funeral practices as well as web search practices. Finally, our research design is constrained by the lack of high frequency historical mortality data.

## Methods

### Monthly-mean adjustment of the mourning and funeral index

For each of the six mourning and funeral search terms, “wreath and elegiac couplet”, “obituary”, “mortuary house”, “cinerary casket”, “cremation” and “pass away” (indexed as *i*), the daily average search volume *V*_*Base*_*p,i,m* during Month *m* in years 2018 and 2019 is the base volume. This period immediately preceded the outbreak of COVID-19. The daily excess monthly-mean adjusted search volume for Province *p* is computed as:

1 $${V}_{Excess}p,i,t=\frac{Vp,i,t-{V}_{Base}p,i,m}{{V}_{Base}p,i,m},$$

where *Vp,i,t* is search volume for Term *i* on Day *t* (of month *m*) in Province *p*.

We take the average of the six indexes to form Province *p*’s aggregate excess mourning and funeral index *V*_*Excess*_*p,t*:

2 $${V}_{Excess}p,t=\frac{1}{6}\sum_{i=1}^{6}{V}_{Excess}p,i,t.$$

We compute the national mourning and funeral index which is the population-weighted average of the thirty-one province-level indexes for each of the six components *V*_*Excess*_*i*,*t* as well as their aggregate *V*_*Excess*_*t*:

3 $${V}_{Excess}i,t=\frac{{\sum }_{p=1}^{31}{V}_{Excess}p,i,t\cdot {Population}_{\mathrm{p},\mathrm{y}}}{{\sum }_{p=1}^{31}{Population}_{\mathrm{p},\mathrm{y}}},$$

4 $${V}_{Excess}t=\frac{{\sum }_{p=1}^{31}{V}_{Excess}p,t\cdot {Population}_{\mathrm{p},\mathrm{y}}}{{\sum }_{p=1}^{31}{Population}_{\mathrm{p},\mathrm{y}}},$$

where *Population*_*p,y*_ is the population for Province *p* at the end of year y. We obtain province-level population data from RESSET. Official population data are available for all provinces up to year 2021. They are only available for some of the provinces in year 2022. For provinces with missing population data 2022, we use their population in 2021. For year 2023, we use the population data of 2022.

For the bulk of our analysis, we use the average of the six terms to form of mourning and funeral index. As can be observed from Panel A of Table S2, among the six terms, the correlation among their search volumes varies considerably, from 0.255 (*p* < 0.01) between “wreath and elegiac couplet” and “obituary” to 0.733 (*p* < 0.01) between “mortuary house” and “cremation”. By averaging the six, we potentially minimize the chance that search volume for a certain term can be endogenously affected by the COVID-19 situations and government initiatives to stem the spread of the virus and that there can be interaction and thus substitution among these terms. Averaging can also help us reduce idiosyncratic volatility associated with specific search terms.

### Excess cases and ratio of mortality

For each province, we compute its daily excess cases of mortality. We first obtain its average annual mortality ratio *Ratio*_*Normal*_*p* based on years 2018 and 2019, before COVID-19. Information on mortality ratio for each province comes from CNRDS (China Research Data Services Platform) database. We multiply this ratio by its population in each year ${Population}_{\mathrm{p},\mathrm{y}}$ and divide it by 365 to obtain the normal daily cases of mortality *D*_*Normal*_*p* for Province *p*:

5 $${D}_{Normal}p=\frac{1}{365}{Ratio}_{Normal}p\cdot {Population}_{\mathrm{p},\mathrm{y}}.$$

Excess cases of mortality for Province *p* on Day *t* is computed as:

6 $${D}_{Excess}p,t={D}_{Normal}p.{V}_{Excess}p,t.$$

The national total excess cases of mortality on Day *t* is the summation of excess cases of mortality of all provinces:

7 $${D}_{Excess}t={\sum }_{p=1}^{31}{D}_{Excess}p,t.$$

We also compute the excess mortality ratio of Province *p Ratio*_*Excess*_*p* during a specific period that we are interested in:

8 $${Ratio}_{Excess}p=\frac{{\sum }_{t=1}^{n}{D}_{Excess}p,t}{{Population}_{\mathrm{p},\mathrm{y}}},$$

where *n* is the number of days during that specific period.

### Checking for out-of-sample validity

We first use the period 2011–2017 to determine if we can estimate the total number of deaths in each province in 2018 and 2019 without significant biases. Total annual search volume *AVp*,*i*,*y* during Year *y* for a particular term *i* in a particular Province *p* is the summation of raw daily search volume for that province during the year.

We take the average of the six indexes to form Province *p*’s aggregate annual mourning and funeral index *AVp,y*:

9 $$AVp,y=\frac{1}{6}\sum_{i=1}^{6}AVp,i,y.$$

For the period 2011–2017, we regress the natural logarithm of annual cases of mortality Ln(*Dp*,*y*) on the natural logarithm of the aggregate mourning and funeral index Ln(*AVp*,*y*) plus a quadratic time trend *Ty* and *Ty*^2^ using the following specification:

10 $$ {\text{Ln}}\left( {Dp,y} \right) \, = \beta_{0} + \beta_{{1}} {\text{Ln}}\left( {AVp,y} \right)) + \beta_{{2}} Ty + \beta_{{3}} Ty^{{2}} + \varepsilon i,y, $$

where *Ty* = (*y* − 2010).

We use Years 2018 and 2019 to determine if there is an out-of-sample estimation bias by first define an estimation error term *EDp*:

11 $$ Error\left( {{\text{Ln}}\left( {Dp_{{{2}0{18}/{2}0{19}}} } \right)} \right) \, = {\text{ Ln}}\left( {Dp_{{{2}0{18}/{2}0{19}}} } \right) \, {-}\widehat{{\beta_{0} }} - \widehat{{\beta_{1} }}{\text{Ln}}\left( {AVp_{{{2}0{18}/{2}0{19}}} } \right) \, {-}\widehat{{\beta_{2} }}T_{{{2}0{18}/{2}0{19}}} {-}\widehat{{\beta_{3} }}T_{{{2}0{18}/{2}0{19}}}^{{2}} , $$

where $\widehat{{\beta }_{0}}$, $\widehat{{\beta }_{1}}$, $\widehat{{\beta }_{2}}$ and $\widehat{{\beta }_{3}}$ are the in-sample estimator for *β*_0_, *β*_1_, *β*_2_ and *β*_3_, respectively. We then compute *t*-values for *EDp*_2018_ and *EDp*_2019_ to determine if there is a bias in our out-of-sample estimation.

### Supplementary Information

Supplementary Information.

## Data Availability

Data are garnered from public sources as indicated in the article. They can be made available to readers after the publication of this article.
