# Supplementary material for: Inferring China’s excess mortality during the COVID-19 pandemic using online mourning and funeral search volume
Source: Sci Rep. 2023 Sep 20;13:15665. doi: 10.1038/s41598-023-42979-1 (PMC10511516; doi:10.1038/s41598-023-42979-1)
Supplement: Supplementary file 1 — Supplementary Information. [file 41598_2023_42979_MOESM1_ESM.docx]

**Supplementary Materials**

Figure S1: Individual search trends for the six Mourning and Funeral related terms, Based on Monthly-Mean Adjustment, during January 1, 2020 to February 28, 2023

**Table S1**

**Excess Cases of Mortality by Provinces Based on Monthly-Mean Adjustment of the Mourning and Funeral Index during December 2022 to February 2023**

| Province | December  2022 | January  2023 | February  2023 | Three Months | Percentage |
| --- | --- | --- | --- | --- | --- |
| Shandong | 42,625 | 25,781 | 3,594 | 72,000 | 10.10% |
| Henan | 42,236 | 21,874 | 6,087 | 70,197 | 9.85% |
| Guangdong | 32,834 | 16,445 | 7,084 | 56,363 | 7.91% |
| Jiangsu | 30,637 | 19,355 | 1,595 | 51,587 | 7.24% |
| Sichuan | 28,183 | 16,739 | 1,385 | 46,307 | 6.50% |
| Hebei | 28,964 | 12,714 | 1,265 | 42,943 | 6.02% |
| Hubei | 21,744 | 13,384 | 2,853 | 37,981 | 5.33% |
| Hunan | 18,883 | 16,717 | 414 | 36,014 | 5.05% |
| Anhui | 17,496 | 11,739 | 170 | 29,405 | 4.12% |
| Zhejiang | 16,759 | 8,914 | -797 | 24,875 | 3.49% |
| Chongqing | 13,451 | 9,256 | 2,121 | 24,827 | 3.48% |
| Guangxi | 11,933 | 6,636 | -663 | 17,906 | 2.51% |
| Beijing | 11,820 | 4,402 | 1,566 | 17,789 | 2.50% |
| Liaoning | 13,295 | 6,090 | -1,716 | 17,669 | 2.48% |
| Jiangxi | 9,351 | 6,639 | 1,648 | 17,637 | 2.47% |
| Shanxi | 8,553 | 6,040 | 1,921 | 16,515 | 2.32% |
| Fujian | 8,918 | 4,647 | 92 | 13,658 | 1.92% |
| Shaanxi | 7,540 | 5,288 | -265 | 12,563 | 1.76% |
| Gansu | 7,838 | 4,162 | 471 | 12,471 | 1.75% |
| Shanghai | 7,332 | 4,132 | 795 | 12,259 | 1.72% |
| Xinjiang | 8,077 | 3,225 | 723 | 12,025 | 1.69% |
| Guizhou | 4,758 | 6,652 | 368 | 11,778 | 1.65% |
| Heilongjiang | 7,003 | 5,613 | -2,177 | 10,439 | 1.46% |
| Jilin | 6,658 | 3,155 | -474 | 9,339 | 1.31% |
| Yunnan | 5,788 | 4,583 | -1,347 | 9,025 | 1.27% |
| InnerMong | 4,714 | 2,402 | -592 | 6,525 | 0.92% |
| Hainan | 3,983 | 2,711 | -221 | 6,473 | 0.91% |
| Tianjin | 3,840 | 1,277 | 48 | 5,165 | 0.72% |
| Qinghai | 2,776 | 1,079 | 627 | 4,483 | 0.63% |
| Xizang | 2,822 | 1,371 | -603 | 3,590 | 0.50% |
| Ningxia | 1,708 | 1,550 | -160 | 3,098 | 0.43% |
|  |  |  |  |  |  |
| Total | 432,519 | 254,573 | 25,813 | 712,905 | 100.00% |

**Table S2**

**Correlations**

Panel A: Correlation among the Baidu Indexes for the Six Mourning and Funeral Terms during 2011-2023

|  | Wreath and Elegiac Couplet | Obituary | Mortuary House | Cinerary Casket | Cremation | Pass Away |
| --- | --- | --- | --- | --- | --- | --- |
| Wreath and Elegiac Couplet | 1 |  |  |  |  |  |
| Obituary | 0.255*** | 1 |  |  |  |  |
| Mortuary House | 0.366*** | 0.658*** | 1 |  |  |  |
| Cinerary Casket | 0.351*** | 0.540*** | 0.675*** | 1 |  |  |
| Cremation | 0.370*** | 0.514*** | 0.733*** | 0.641*** | 1 |  |
| Pass Away | 0.274*** | 0.679*** | 0.673*** | 0.619*** | 0.602*** | 1 |

Panel B: Pairwise Correlation between Annual Cases of Mortality and Annual Baidu Search Volume for the Six Mourning and Funeral Related Phrases in 31 Provinces during 2011 to 2019

|  | Cases of Mortality |
| --- | --- |
| Wreath and Elegiac Couplet | 0.644*** |
| Obituary | 0.482*** |
| Mortuary House | 0.574*** |
| Cinerary Casket | 0.603*** |
| Cremation | 0.615*** |
| Pass Away | 0.433*** |
|  |  |
| Average Index | 0.578*** |

**Table S3**

**Excess Mortality Ratio and the Change in Mortality Ratio from Its Normal Level by Provinces, Based on Monthly-Mean Adjustment of the Mourning and Funeral Index, during December 2022 to February 2023**

|  | December 2022 | | | January 2023 | | | February 2023 | | | Three Months | | |
| --- | --- | --- | --- | --- | --- | --- | --- | --- | --- | --- | --- | --- |
|  | Ratio | s.e. | % Change | Ratio | s.e. | % Change | Ratio | s.e. | % Change | Ratio | s.e. | % Change |
| Anhui | 0.000286 | 0.000030 | 58.04% | 0.000192 | 0.000023 | 38.94% | 0.000003 | 0.000010 | 0.56% | 0.000481 | 0.000053 | 32.51% |
| Beijing | 0.000540 | 0.000082 | 118.72% | 0.000201 | 0.000033 | 44.21% | 0.000072 | 0.000010 | 15.73% | 0.000813 | 0.000108 | 59.55% |
| Chongqing | 0.000419 | 0.000047 | 67.41% | 0.000288 | 0.000036 | 46.39% | 0.000066 | 0.000022 | 10.63% | 0.000773 | 0.000076 | 41.48% |
| Fujian | 0.000213 | 0.000028 | 42.14% | 0.000111 | 0.000025 | 21.96% | 0.000002 | 0.000010 | 0.44% | 0.000326 | 0.000047 | 21.51% |
| Gansu | 0.000315 | 0.000022 | 57.16% | 0.000167 | 0.000020 | 30.35% | 0.000019 | 0.000010 | 3.43% | 0.000501 | 0.000048 | 30.32% |
| Guangdong | 0.000259 | 0.000034 | 69.91% | 0.000130 | 0.000026 | 35.02% | 0.000056 | 0.000006 | 15.08% | 0.000444 | 0.000050 | 40.00% |
| Guangxi | 0.000236 | 0.000031 | 47.55% | 0.000131 | 0.000017 | 26.44% | -0.000013 | 0.000018 | -2.64% | 0.000355 | 0.000050 | 23.78% |
| Guizhou | 0.000124 | 0.000025 | 21.78% | 0.000173 | 0.000026 | 30.45% | 0.000010 | 0.000015 | 1.68% | 0.000306 | 0.000044 | 17.97% |
| Hainan | 0.000390 | 0.000069 | 78.36% | 0.000266 | 0.000045 | 53.35% | -0.000022 | 0.000033 | -4.35% | 0.000634 | 0.000103 | 42.45% |
| Hebei | 0.000389 | 0.000037 | 75.70% | 0.000171 | 0.000021 | 33.23% | 0.000017 | 0.000007 | 3.31% | 0.000577 | 0.000064 | 37.41% |
| Heilongjiang | 0.000224 | 0.000046 | 40.67% | 0.000180 | 0.000030 | 32.59% | -0.000070 | 0.000016 | -12.64% | 0.000334 | 0.000070 | 20.21% |
| Henan | 0.000427 | 0.000039 | 76.24% | 0.000221 | 0.000030 | 39.48% | 0.000062 | 0.000010 | 10.99% | 0.000710 | 0.000068 | 42.24% |
| Hubei | 0.000373 | 0.000048 | 64.46% | 0.000230 | 0.000031 | 39.67% | 0.000049 | 0.000011 | 8.46% | 0.000651 | 0.000070 | 37.53% |
| Hunan | 0.000285 | 0.000032 | 48.32% | 0.000252 | 0.000032 | 42.78% | 0.000006 | 0.000015 | 1.06% | 0.000544 | 0.000061 | 30.72% |
| InnerMong | 0.000196 | 0.000028 | 41.17% | 0.000100 | 0.000024 | 20.98% | -0.000025 | 0.000011 | -5.17% | 0.000272 | 0.000048 | 18.99% |
| Jiangsu | 0.000360 | 0.000049 | 62.30% | 0.000228 | 0.000041 | 39.36% | 0.000019 | 0.000011 | 3.24% | 0.000607 | 0.000077 | 34.96% |
| Jiangxi | 0.000207 | 0.000024 | 41.56% | 0.000147 | 0.000022 | 29.51% | 0.000036 | 0.000010 | 7.32% | 0.000390 | 0.000040 | 26.13% |
| Jilin | 0.000280 | 0.000044 | 51.83% | 0.000133 | 0.000031 | 24.56% | -0.000020 | 0.000018 | -3.69% | 0.000393 | 0.000068 | 24.23% |
| Liaoning | 0.000314 | 0.000058 | 52.25% | 0.000144 | 0.000031 | 23.93% | -0.000041 | 0.000012 | -6.74% | 0.000418 | 0.000081 | 23.15% |
| Ningxia | 0.000236 | 0.000050 | 51.05% | 0.000214 | 0.000051 | 46.33% | -0.000022 | 0.000046 | -4.79% | 0.000427 | 0.000091 | 30.87% |
| Qinghai | 0.000467 | 0.000103 | 92.08% | 0.000181 | 0.000050 | 35.80% | 0.000105 | 0.000062 | 20.81% | 0.000753 | 0.000137 | 49.56% |
| Shaanxi | 0.000191 | 0.000026 | 37.06% | 0.000134 | 0.000025 | 25.99% | -0.000007 | 0.000010 | -1.30% | 0.000318 | 0.000045 | 20.58% |
| Shandong | 0.000419 | 0.000050 | 69.47% | 0.000253 | 0.000032 | 42.02% | 0.000035 | 0.000012 | 5.86% | 0.000708 | 0.000077 | 39.12% |
| Shanghai | 0.000295 | 0.000039 | 65.75% | 0.000166 | 0.000032 | 37.05% | 0.000032 | 0.000007 | 7.13% | 0.000492 | 0.000060 | 36.64% |
| Shanxi | 0.000246 | 0.000025 | 53.53% | 0.000174 | 0.000021 | 37.81% | 0.000055 | 0.000010 | 12.02% | 0.000474 | 0.000041 | 34.46% |
| Sichuan | 0.000337 | 0.000047 | 58.10% | 0.000200 | 0.000033 | 34.50% | 0.000017 | 0.000009 | 2.86% | 0.000553 | 0.000070 | 31.82% |
| Tianjin | 0.000280 | 0.000048 | 63.49% | 0.000093 | 0.000027 | 21.11% | 0.000003 | 0.000014 | 0.79% | 0.000376 | 0.000067 | 28.46% |
| Xinjiang | 0.000312 | 0.000024 | 84.25% | 0.000125 | 0.000019 | 33.64% | 0.000028 | 0.000016 | 7.55% | 0.000464 | 0.000050 | 41.81% |
| Xizang | 0.000771 | 0.000191 | 207.54% | 0.000375 | 0.000116 | 100.82% | -0.000165 | 0.000052 | -44.34% | 0.000981 | 0.000259 | 88.01% |
| Yunnan | 0.000123 | 0.000023 | 23.99% | 0.000098 | 0.000021 | 18.99% | -0.000029 | 0.000013 | -5.58% | 0.000192 | 0.000039 | 12.47% |
| Zhejiang | 0.000256 | 0.000035 | 56.18% | 0.000136 | 0.000031 | 29.88% | -0.000012 | 0.000010 | -2.67% | 0.000380 | 0.000059 | 27.79% |
|  |  |  |  |  |  |  |  |  |  |  |  |  |
| National | 0.000306 | 0.000034 | 52.21% | 0.000180 | 0.000026 | 30.75% | 0.000018 | 0.000007 | 3.12% | 0.000505 | 0.000057 | 28.69% |

**Table S4**

**Regression of Natural Logarithm of Annual Cases of Mortality on the Natural Logarithm of the Aggregate Mourning and Funeral Index during 2011-2019**

|  | Ln(*Dp*,*y*) |
| --- | --- |
| Ln(*AVp*,*y*) | 1.020*** |
|  | (8.11) |
| *Ty* | -0.270*** |
|  | (-5.97) |
| *Ty*^2^ | 0.015*** |
|  | (4.87) |
| Constant | 2.756** |
|  | (2.45) |
|  |  |
| Observations | 279 |
| Adj. R-squared | 0.657 |

***, ** and * indicate significance levels at 1%, 5% and 10% respectively.
